# The etiopathogenesis of uterine fibromatosis

**Published:** 2016

**Authors:** L Manta, N Suciu, O Toader, RM Purcărea, A Constantin, F Popa

**Affiliations:** * Department of Gynecology and Obstetrics, “Gh. Polizu” Maternity, “Alfred Rusescu” Mother and Child Care Institute, Bucharest, Romania; ** Department of Internal Medicine, “Carol Davila” Hospital, Bucharest, Romania; *** Department of General Surgery, “Sf. Pantelimon” Clinical Hospital, Bucharest, Romania

**Keywords:** uterine fibromatosis, uterine fibroids, uterine leiomyomas, benign tumorsn

## Abstract

Uterine fibroids or uterine leiomyomas are the most common benign tumors of the uterus among women of fertile age, while the etiology is still incompletely elucidated. The occurrence and development of the fibromatosis may be related to certain risk factors and genic mechanisms, although the exact causes are not yet fully known. The development of uterine fibroids is correlated not only with the metabolism and with the level of female sexual hormones, estrogen, and progesterone, but also with the number of these hormone receptors expressed on the surface of the myometrium. Proliferative effects of estrogen and progesterone may be exercised through proinflammatory factors (TNF alpha), growth factors (IGF1, IGF2, TGFbeta3 and betaFGF) or inhibitors of apoptosis (p53 suppression). A number of predisposing factors such as ethnicity – black skin, early menarche, nulliparity, caffeine and alcohol, chronic inflammation, obesity, were also identified. Approximately 40% of the uterine fibroids are caused by the same cytogenetic alterations found in the other tumor types such as kidney, lung, or leiomyosarcoma. As part of a system dysfunction, uterine fibromatosis was connected to other disorders such as AHT (arterial hypertension), endometrium adenocarcinoma, adenomyosis, endometriosis, diabetes mellitus, breast tumors, seemingly with a common causality. The action and effect of some hormonal imbalances over the various organs depend on the histological and local expression particularities of the various receptors, being the cause for many disorders, among which the uterine fibromatosis, coexisting or accompanying the later. This article examines and summarizes the latest data refreshed literature etiopathogenesis offering indicators of uterine fibroids.

## Introduction

The uterine fibroids or the leiomyomas are the most common benign tumor of the uterus in fertile women, their etiology being yet not fully understood. These tumors are generated by the proliferation and transformation of the myometrial tissue in certain physiopathological conditions. With the development of the ultrasonography, the prevalence of uterine fibromatosis increased from approximately 35% (exclusively clinical diagnosis) to approximately 50% in fertile women (ultrasonographical diagnosis).

The occurrence of the uterine fibromatosis increases up to approximately 80% on the hysterectomy specimens [**1**].

The occurrence and development of fibromatosis may be related to certain risk factors and genic mechanisms, although the exact causes are not yet fully known.

## Risk factors in the uterine fibromatosis

### 1. The estrogens and progesterone

The development of the uterine fibroma was linked, in many studies, with the metabolism and the level of feminine sexual hormones, respectively the estrogen and the progesterone. The growth, stagnation or the regression of uterine fibroma in various women, apparently with similar hormonal levels, led to the idea of a monoclonal tumoral origin, with particular molecular and surface receptors’ characteristics [**2**,**3**].

The explanation may be the fact that the development of leiomyoma is related not only to the estrogen level, but also to the number of expressed receptors on the myometrial surface. Thus, the expression of the receptors over the α- and β-type estrogens was found to be higher at the tumoral level. Due to the epigenetic changes of the micro-ARN, a hypomethylation of the estrogenic receptors with their activation occurs at the tumoral level. The proliferative effect of the estrogens manifests through proinflammatory factors (cytokine), growth factors (IGF1, IGF2, TGFbeta3 and betaFGF) and cellular apoptosis inhibitors (suppression p53).

The quick growth of the fibroma after the age of 30 and especially during premenopause showed a relation between these and the age related changes in the hormonal constellation (fluctuations) – the change of the estroprogestative balance, which implies an increasing LH level during perimenopause, leading to a growth in volume of the uterine fibroma [**4**]. 

The progesterone activity manifests through its A and B receptors. At the fibroma level, the number of progesterone receptors was found to be elevated. The mitogenic effects of the progesterone result in an increase in the levels of IGF, TGF beta 3 and a decrease in the alpha TNF expression. The uterine fibroma regression under treatment with anti-progesterone (RU486) or with progesterone receptor blockers (ulipristal acetate), prove the progesterone’s proliferative effect.

### 2. Ethnicity

As it is already known, belonging to the black race is a risk factor for the uterine fibromatosis. Wey et al. discovered a different expression, depending on the ethnic belonging of the protein coding genetic mutations at the leiomyoma levels, which leads to a different progression of this disorder in different races [**5**].

### 3. Early menarche

The early menarche, before the age of 10 years, proved to be a risk factor, while the onset of menarche after 16 years seems to be a protective factor against the development of the uterine fibromatosis. The uterine menarche is a risk factor for endometrium or breast cancer, diseases to which the uterine fibromatosis may be associated with [**6**].

### 4. The parity and the pregnancy

The parity is in a reverse relationship with the occurrence of uterine fibromatosis. The protective mechanism of the pregnancy is yet unknown, but small lesions may appear during the uterine regression and reshaping during confinement, due to the apoptosis processes occurring at this time [**7**,**8**].

Apparently, the uterine fibromas are sensitive to the uterine ischemia during childbirth, as well as to the uterine reshaping during confinement, thus leading to their involution.

### 5. The caffeine and alcohol consumption

Recent studies showed a direct proportional relationship between the caffeine and alcohol consumption and the occurrence of uterine fibromatosis. The consumption of more than 500 µg of caffeine/ day was associated with an increased risk of developing uterine fibromatosis [**9**].

### 6. Chronic inflammation

The proposed mechanism to explain the causality between the inflammation and tumor proliferation is the injury caused by the infection, which causes the growth of the extracellular matrix, cellular proliferation due to pro-inflammatory and growth factors, decreasing apoptosis and abnormal tissue repair [**10**,**11**].

### 7. Hormonal therapiesn

Until now, the hormone substitution therapy could not be incriminated for the volume growth of the uterine fibroids. The influence of oral contraceptives administration in the onset and development of uterine fibroma has remained a matter of controversy so far, the effects being probably directly proportional with the dosage and the type of estrogen and progestin from the tablet composition. The risk of uterine fibromatosis onset and bleeding seems to be reduced strictly during the administration of the oral contraceptives.

Some studies showed an up to 30% risk decrease in the onset of uterine fibromatosis in women with normal weight who have been using oral contraceptives for more than 10 years, probably due to prolonged ovarian inhibition while also avoiding the fluctuation and hormonal changes specific to the various reproductive time intervals. Thus, even if the oral contraceptives do not decrease the size of the tumors, they may reduce the symptomatology and the bleeding, without any contraindications in women with uterine fibroma [**12**].

### 8. Smoking

Smoking reduces the risk of uterine fibromatosis onset. Nicotine inhibits the aromatase, decreases the estrogen hormones levels both by ovarian follicular apoptosis, and by decreasing the conversion rate of androgens to estrogens. Also, nicotine changes the estradiol metabolism by hydroxylation and decreases the tissular bioavailability.

### 9. Genetic factors

Until now, uterine fibromatosis was not viewed as a disorder with a genetic component. Recent findings proved the importance of cytogenetic changes in the etiopathogenesis of the uterine fibromatosis. Thus, approximately 40% of the uterine fibroma are caused by common cytogenetic alterations with other types of tumors, such as renal, pulmonary or leiomyosarcoma [**13**,**14**].

Among the mutations responsible for causing uterine fibromatosis are the translocations between chromosomes 12 and 14, translocations between chromosomes 6 and 10, deletions of chromosomes 3 and 7 [**15**].

The HMGA2 found in translocations 12:14 seems to be the most frequent cytogenetic anomaly, occurring in approximately 20% of the uterine fibroma, but not in the normal myometrium. The HMGA2 gene codes proteins responsible for embryo-type proliferation, which act at the cellular DNA level. This gene is expressed at the leiomyomatous cell level, but in other tissues too, such as in the lung or liver tissues. The epigenetic changes (RNA) were also associated with the pathogenesis of uterine fibromatosis. A hypermethylation of the alpha estrogen receptors was observed in the fibroma, at the cellular level. The protein and enzyme hypermethylation phenomenon was globally associated with the development and progression of the uterine fibromatosis. The DNA and protein methylation is a mechanism that regulates the activity of transcription and the enzymatic activity inversely proportional to its intensity. Thus, the cellular DNA hypomethylation leads to an increased transcriptional activity and cellular proliferation specific to the tumoral development (epigenetic changes). It seems that the molecular changes responsible for the apparition of uterine fibromatosis occur during childhood. MicroRNA is a small molecule, without any role in the genetic information coding, which functions in the cellular RNA hipoexpression (silencing) and post-transcriptional regulation of the gene expression. Several microRNA molecules, such as let7 (miR21, miR93, miR106b, miR200) have an altered function in the uterine fibromatosis. Additional studies are needed for future identification and targeted gene therapy of the uterine fibromatosis [**16**,**17**].

### 10. Obesity

Obesity is considered a risk factor for the uterine fibromatosis. Various studies reported a BMI higher than 25 to almost 70% in female patients with fibromatosis [**18**,**19**].

The explanation is the increased peripheral estrogen production in the adipose tissue, through aromatization. Like the hyperplasia and endometrial adenocarcinoma, the uterine fibroma are considered tumors whose development depends on the high estrogenic level specific to the obesity.

### 11. Bleeding physiopathology in the uterine fibromatosis (myometrial junction)

The subendometrial myometrium (myometrial junction) is a distinct hormone-dependent distinct compartment that may be seen in echography and MR scans. Although histologically identical to the rest of the myometrium, the junction area has a different structural and functional behavior. According to their location in the thickness of the uterine wall, fibromas are classified as submucous, intramural, and subserous. Submucous fibromas are considered those that reshape or protrude the uterine cavity. Submucous fibromas are associated with metrorrhagia and reproductive dysfunction [**20**,**21**,**22**].

The size and level of the protrusion in the uterine cavity are the main factors determining the severity of the symptoms. Usually, the origin of the fibroma inside the cavity is at the level of the internal myometrium (myometrial junction), but the voluminous fibroma originating in the external myometrium may also cause the deformity of the uterine cavity. The fibromas occurring in the junction area have a faster growth, express more estrogen and progesterone receptors compared to the fibromas from the rest of the myometrium, a fact that may be used in the future for the identification of the origin area and for the disorder’s evolution prognosis. The junction area may be identified in echography as a hypoechogenic, linear image, located in the subendometrium.

The fibromas with submucous origin cause discontinuities in the MR or echographic image of the junction area, while the fibromas in the rest of the myometrium only displace it and cause deformation by pushing, without changing its morphology. Identifying the fibromas’ origin area might be useful in counseling and explaining the prognosis and disorder’s evolution of the female patients. A thickening of the junction area might be observed in female patients with fibromatosis, in whom vascular and biochemical changes occur, favoring the bleeding at the endometrial level and decreasing the embryo implantation rate. The submucous fibromas are also generated in this area. These changes should be separated and differentiated from the adenomyosis. The studies showed a decrease in the success rate of the assisted reproduction techniques in the case of thickening or discontinuities in the junction area.

The mechanism of myometrial differentiation is not yet completely known, but it seems to occur under the influence of the estrogen hormones, mediated by the cytokines and uterotonins locally released by the basal endometrium and the endomyometrial lymphocytes. The thickening of the junction area, i.e. the hyperplasia, is a frequent MR finding in women with a menstrual dysfunction and metrorrhagia.

### 11. Associated dysfunctions, mechanism of action

As part of a system dysfunction, uterine fibromatosis is connected to other disorders such as AHT (arterial hypertension), endometrium adenocarcinoma, adenomyosis, endometriosis, mellitus diabetes, breast tumors, seemingly with a common causality.

Arterial hypertension increases the risk of uterine fibromatosis by a similar mechanism to that found in atherosclerosis, which induces proliferation in the vascular smooth muscle (proatherogenic). The hemodynamic stress caused by AHT causes lesions in the vascular endothelium with a consequent dysfunction, respectively increased permeability, smooth muscular cell migration and forming the atheroma patches and the fibrous tissue. The same mechanism that initiates the fibromas formation seems to cause lesions in the myometrial cells. The uterine fibromas are generated in the myometrium, uterine arteries, or uterine conjunctive tissue. Findings concerning the structure and uterine vascularization function anomalies in the fibromatosis suggest their common causality. The atheroma patches and the uterine fibromas have similar characteristics, among which the monoclonal origin, the similar behavior in cellular cultures; in preeclampsia the myometrial cells accumulate lipids in the same manner as the atherosclerotic cells, with a common tendency in fibrosis and calcification. These findings create a temporal connection, in direct proportion, between the AHT level and the uterine fibroma, also connecting the proatherogenic impact of AHT with the reproductive health.

Although it may be considered that the hyperinsulinemia and the increased level of IGF 1 might lead to and favor the proliferation and development of leiomyoma, it seems that there is a negative association of the fibroma with diabetes mellitus, the latter rather being a protective factor, probably due to the angiopathy [**23**].

In various studies, it was observed that the uterine fibromatosis coexists in proportion of up to 20% with the endometriosis, especially in symptomatic disorders, having a common etiopathogenesis and being independently involved in the infertility mechanisms [**24**].

The progesterone receptors mediate the ovary secreted progesterone action and, along with the estrogens, prepare the endometrium for implantation, keeping the pregnancy, differentiation, and maturation of the mammary tissue. The exact identification of the way each feminine hormone acts remains a challenge. The pathologies of the uterus and breast, including the endometrium cancer, endometriosis, uterine fibromatosis, and breast cancer are in direct relationship with the blood estrogen level, which is considered a mitogenic factor. Numerous findings also supported the distinct role of progesterone in the genesis of these disorders. Progesterone antagonizes the estrogen-induced endometrial proliferation, and therefore its insufficiency substantially increases the risk of endometrium cancer. In endometriosis, both the eutopic and the ectopic endometrial tissue have a weak response to progesterone and are considered progesterone-resistant, which adds to the evolution of the disorder. Progesterone induces tumor development and growth in the uterine fibromatosis by increasing the tumor proliferation, cellular hypertrophia and growth in the deposition of the extracellular matrix. Progesterone is a proliferative and carcinogenetic factor in the normal and neoplastic mammary tissue. A key mechanism for the progesterone-differentiated action in these tissues might be the paracrine interaction of the progesterone receptors in the stroma and the epitelium. The normal endometrium is a mucosa with stromal cells that expresses a high quantity of progesterone receptors, which influence the cell proliferation and differentiation, having an anti-carcinogenetic role. The main cellular targets of progesterone in the mammary and fibromatous tissue, as opposed to the endometrial tissue, are the mammary and leiomyomatous epithelial cells, and the stroma with increased progesterone receptors expression lacks almost completely, therefore, the stroma’s protective, and antiproliferative effect.

The action and effect of some hormonal imbalances over the various organs depend on the histological and local expression particularities of the various receptors, being the cause for many disorders, among which the uterine fibromatosis, coexisting or accompanying the latter.
